# Analysis of growth-phase regulated genes in *Streptococcus agalactiae *by global transcript profiling

**DOI:** 10.1186/1471-2180-9-32

**Published:** 2009-02-10

**Authors:** Izabela Sitkiewicz, James M Musser

**Affiliations:** 1 Center for Molecular and Translational Human Infectious Diseases Research, The Methodist Hospital Research Institute, Houston, Texas 77030, USA; 2 Department of Pathology, The Methodist Hospital, Houston, Texas 77030, USA; 3 National Medicines Institute, Dept. of Epidemiology and Clinical Microbiology, Chelmska 30/34, 00-725 Warsaw, Poland

## Abstract

**Background:**

Bacteria employ multiple mechanisms to control gene expression and react to their constantly changing environment. Bacterial growth in rich laboratory medium is a dynamic process in which bacteria utilize nutrients from simple to complex and change physical properties of the medium, as pH, during the process. To determine which genes are differentially expressed throughout growth from mid log to stationary phase, we performed global transcript analysis.

**Results:**

The *S. agalactiae *transcriptome is dynamic in response to growth conditions. Several genes and regulons involved in virulence factor production and utilization of alternate carbon sources were differentially expressed throughout growth.

**Conclusion:**

These data provide new information about the magnitude of plasticity of the *S. agalactiae *transcriptome and its adaptive response to changing environmental conditions. The resulting information will greatly assist investigators studying *S. agalactiae *physiology and pathogenesis.

## Background

Bacteria employ multiple mechanisms to control gene expression and react to their constantly changing environment. These processes are especially critical for bacterial pathogens to survive and cause disease in humans and other hosts. Global control of gene expression is achieved using alternative sigma factors, two-component systems (TCSs), small regulatory RNAs, regulators such as RelA and LuxS, or concerted action of regulons (for a review see [1-6] and references therein). Gram positive pathogens such as group A *Streptococcus *(*S. pyogenes*, GAS) and group B *Streptococcus *(*S. agalactiae*, GBS) lack (or have limited number) of alternative sigma factors of fully confirmed function [7-9]. Analyses of global transcription in GAS under various growth conditions including saliva, blood, and tissue has shown that environmental response regulation is achieved using other mechanisms such RNA stability [10], "stand alone" regulators such as *mga *[11], or TCSs [12-15]. These transcriptome analyses have been especially useful in providing new information about microbial physiology and leads for pathogenesis research. However, the transcriptional response of GBS to changing growth conditions has not been fully analyzed, only single reports were recently published [16]. GBS is an important human and cow pathogen, responsible for thousands of severe invasive infections in man and large economic loss attributable to bovine mastitis (see [17,18] and references therein).

One of the best examples of sequential gene regulation is bacterial growth in complex medium and activation of stationary phase genes. During growth, bacteria utilize available nutrients, presumably from simple to more complex, and alter their environment (e.g. decrease or increase in pH) as a result of metabolic byproduct release. Therefore, stationary phase can be considered the acid/alkali stress, depending on the type of metabolism and nutrients utilized. GAS grown to stationary phase sequentially expresses genes involved in various aspects of GAS physiology, metabolism and virulence, many genes activated or repressed during the transition to stationary phase have also been shown to play a role in GAS virulence [19]. The purpose of the present study was to identify growth phase-regulated genes in GBS, with a special interest in providing new information about virulence factor gene expression.

## Methods

### Sample collection for microarray analysis

**Figure 1:**
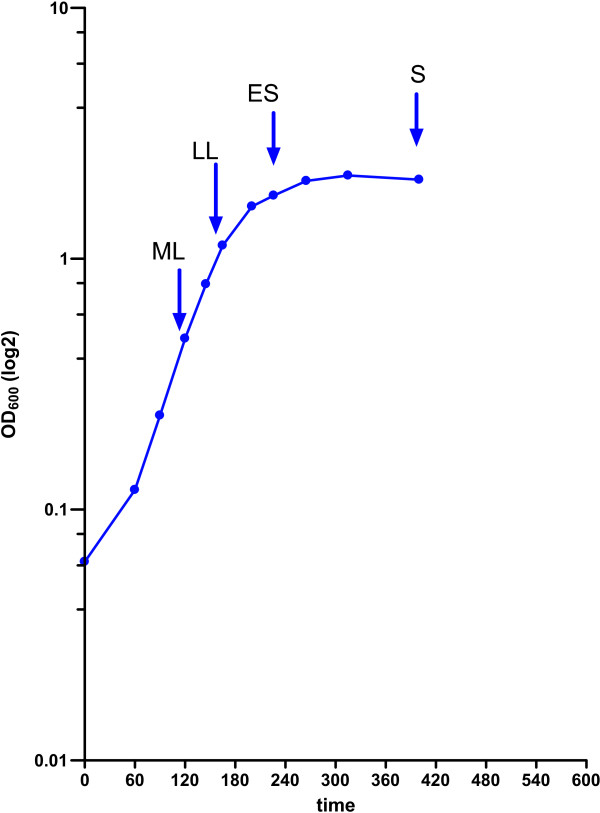
**Growth curve of NEM316 in THY medium**. Arrows denote points of sample collection.

GBS strain NEM316 [7] was grown as three static cultures (3 biological replicates) in liquid Todd Hewitt medium with 0.5% yeast extract in the 5% CO_2 _atmosphere at 37°C [12]. Samples were collected at OD_600 _approximately 0.5, 1.0, 2.0 and 2.5, representing mid-logarithmic (ML), late-logarithmic (LL), early stationary (ES) and stationary (S, about 3 h from entering the phase) growth phases, respectively. Growth curve of bacterial cultures used for data collection is presented as Figure 1. Five ml of each sample were immediately mixed after collection with 10 ml of RNAProtect (Qiagen), centrifuged and stored at -80°C until processing.

Glucose content of the medium at the beginning and end of the culture was measured using Optium Xido glucometer (Abbot) and pH was checked using pH test strips (Macherey Nagel).

### RNA isolation

GBS cells were mechanically opened by shaking with glass beads (Lysing Matrix B, MPBio) and TRIZOL (Invitrogen). RNA was isolated according to Chomczynski and Sacchi [20], with an additional purification step using RNeasy columns (Qiagen). Targets for hybridization with the array were prepared according to array manufacturer (Affymetrix) as described previously [12].

### Array hybridization and data acquisition

The custom expression array manufactured by Affymetrix [21] contained redundant sets of probes representing 1,994 open reading frames (ORFs) of previously sequenced GBS strain NEM316 [7]. Arrays were hybridized and scanned according to the manufacturers instructions. The results of hybridization were normalized to mean of total intensity of GBS probes to allow multiple time point comparison. Array hybridization results are presented as Additional file 1 and are deposited in GEO database http://www.ncbi.nlm.nih.gov/projects/geo/ under GSE12238 accession number.

## Results and Discussion

### General trends in transcription

**Figure 2:**
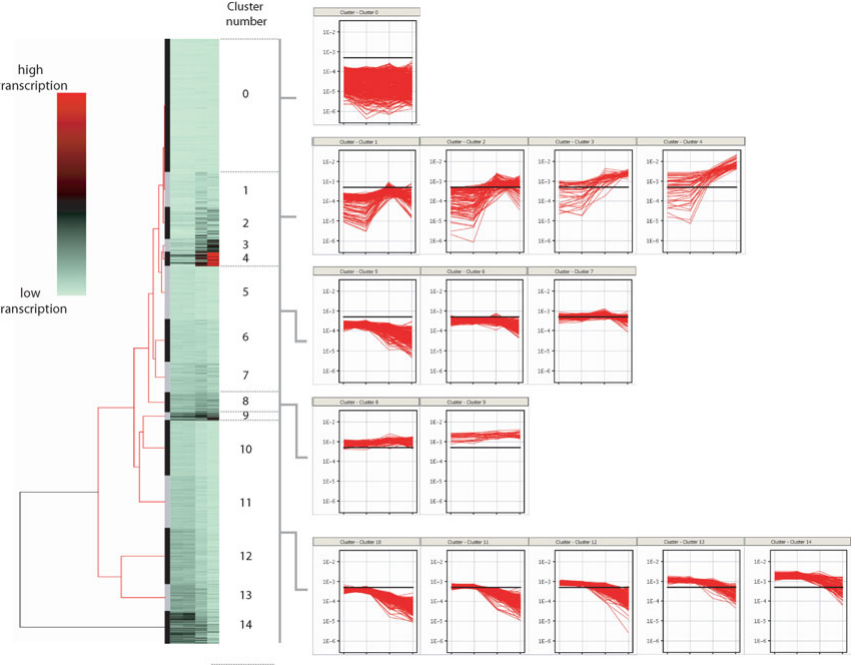
**Grouping of *S. agalactiae *transcripts into distinct 15 clusters based on expression profiles from ML to S growth phases**. The dendrogram and clusters were generated using a self organizing map algorithm and represent changes in expression of 1,994 transcripts at four consecutive time points: ML, LL, ES, and S phases. Cluster 0 genes had low level of transcription. Clusters 1–4 genes positively correlated with stationary phase of growth transcription level and peaked in the ES (clusters 1 and 2) or S (clusters 3 and 4) phase of growth. Clusters 5 and 10–14 are negatively correlated with the S phase of growth; transcription of genes grouped in these clusters reached their peak in ML phase and decreased in S phase. Genes in clusters 6–9 are expressed relatively steadily during growth although at various levels of expression, ranging from very high (cluster 9) to mid-low (cluster 6). The black horizontal line on the cluster graphs represents average transcription level of the complete dataset. The transcript level in each cluster is plotted using a logarithmic scale. Number of transcripts in clusters: Cluster 0, 440; Cluster 1, 115; Cluster 2, 106; Cluster 3, 42; Cluster 4, 47; Cluster 5, 175; Cluster 6, 140; Cluster 7, 100; Cluster 8, 66; Cluster 9, 26; Cluster 10, 183; Cluster 11, 173; Cluster 12, 185; Cluster 13, 89; Cluster 14, 107.

After determining transcript levels for all probe sets, the 1,994 transcripts were grouped into 15 clusters based on their behavior during growth (Figure 2) (self organizing map algorithm; Array Assist 5.1.0 package, Stratagene). The clusters were grouped into five main categories. The first 3 categories contain genes whose transcription did not correspond to growth phase, and were either expressed at low (cluster 0), medium (clusters 6, 7), or high (clusters 8, 9) levels in all phases of growth. Category 4 genes (clusters 1–4) exhibited increased transcription in ES or S phase, and category 5 genes (clusters 5, 10–14) had transcription levels that peaked in ML phase and decreased into S phase.

### Genes exhibiting growth phase-independent transcription

Genes in clusters 6, 7, 8, and 9 did not show growth phase-dependent transcriptional regulation. The genes are clustered instead based on their transcript level and general profile. Clusters 6 and 7 contain genes that are expressed at the same level until ES phase to slightly lower expression in S phase. Clusters 8 and 9 contain genes, which the transcript level is steady or slightly increases over time. Cluster 9 is especially interesting in that it contains a group of highly expressed genes that includes *fabF, fabZ, fabH*, and *accBCD *(gbs0331, 0336–0341) encoding subunits of beta-ketoacyl-ACP synthase, subunits of acetyl-CoA carboxylase, 3-hydroxydecanoyl-ACP dehydratase, and biotin carboxylase. Other genes in cluster 9 involved in energy production are ATP synthase subunits (*atpABEF*, gbs 0875–7 and 9). Interestingly, cluster 9 contains a transcript of putative catabolite control protein A (*ccpA*), and the amount grows steadily to increase about three-fold in S phase in comparison with ML (Table 1). CcpA is a major mediator of carbon catabolite repression – the control mechanism of nutrient utilization. In GAS, CcpA has recently been shown to be a critical direct link between carbohydrate utilization and virulence [21]. Function of CcpA in GBS has been not experimentally confirmed yet. Based on the consensus CcpA binding site (*cre *sequence), we detected that genome of NEM316 strain contains multiple putative *cre *sites in promoter sequences of multiple genes (Table 2), what might be correlated with changes in expression of genes involved in arginine and carbohydrate metabolism (see below). The transcript encoding HPr carrier protein, another element of the CcpA regulatory pathway in Gram-positive bacteria, also belongs to cluster 9. HPr kinase, however, is an S phase-related gene (see below).

**Table 1:** Fold changes in transcript levels of GBS genes.

Gene	Fold change in S phase (S/ML ratio)	Putative function
**S phase related genes**		

*hrcA, grpE*, *dnaK *(gbs0094–96),	+4 to +7.5	Stress response

*clpE*, and *clpL *(gbs0535 and gbs1376)	+4.5 and +7.5	Chaperones

gbs1202/1204, gbs1721, gbs1772	+ 30 to +64	Putative stress response proteins from Gls24 and universal stress response families

gbs2083–2085	+350 to over +1000	arginine/ornithine antiporter, carbamate kinase, ornithine carbamoyltransferase

gbs2122–2126	+55 to +150	arginine deiminase ornithine carbamoyltransferase, arginine/ornithine antiporter carbamate kinase

*glpKDF *(gbs0263–5)	+45 to +63	putative operon responsible for glycerol uptake and utilization.

**Nutrient utilization and energy metabolism**		

*fba *gbs0125	+2.2	fructose-bisphosphate aldolase

*plr *gbs1811	+3.1	glyceraldehyde 3P-dehydrogenase

*pgk *gbs1809	+2.8	phosphoglycerate kinase

*eno *gbs0608	+2.5	enolase

*acoAB *(gbs 0895–0896)	+4	pyruvate dehydrogenase

*ldh *gbs0947	+2.8	L-lactate dehydrogenase

**Regulators and signal transduction systems**		

gbs 1671/2	-2	TCS CovR/S

gbs1908/9	+10/14	TCS, homolog of GAS Spy1106/7 (SF370)

gbs1934/5	+5/+5	TCS, homolog of Spy1061/2 (SF370)

gbs2081/2	-2.3/-1.7	TCS, putative arginine utilization regulator

gbs2086/7	2.5/2.6	TCS, putative arginine utilization regulator

gbs1834/5	-7.5/-11.7	TCS

gbs1397/8	-7/-5.8	TCS

gbs0597/8	-5/-8.5	TCS

gbs0121/2	-2/1	TCS

gbs0298/9	-3/-1.8	TCS

gbs0309/10	-3.3/-3	TCS

gbs0429/30	-2.4/-1.6	TCS

gbs0963/4	+1.7/+2	TCS

gbs1019/20	-1.9/-1.9	TCS

gbs1947/8	-3/-2.4	TCS

gbs1943/4	-2.1/-2.7	TCS

gbs0680	+3.1	CcpA

gbs0191	+50	putative transcriptional antiterminator of the BglG family

gbs0469	-34	Regulator of unknown function

*relA *(gbs1928)	-50	GTP pyrophosphokinase

*codY *(gbs1719;)	-8	Global regulator

*luxS *(gbs0294)	-3	Quorum sensing

*mecA *(gbs0135)	-20	Global regulator of competence

**Virulence factors**		

gbs1420	+6.26	choline-binding protein

gbs1539	+4.67	cell wall anchored protein

gbs1929	+5.48	putative nucleotidase

gbs1143	+2.61	cell wall anchored protein

gbs0451	-2	paralog of C5A peptidase precursor

gbs1104	-6.15	cell wall anchored protein

gbs1529	-11	putative fibronectin binding protein

gbs0850	-3	putative fibronectin binding protein

gbs1307	-3	laminin binding protein

gbs1926	-3	putative laminin binding protein

gbs1475/6	-5.5	sortases

gbs0644–0654	-1.6 to -2.8	hemolysin

gbs1061–1076	-2.5 to -12.9	pathogenicity island IX

gbs1233–1247	-3 to -12.4	capsule synthesis

*cpsX *gbs1250	4.4	capsule synthesis regulator

gbs1478/9, gbs1481, gbs1484/5, gbs1492–1494	-3 to -12	Putative group B antigen

*Cfa *gbs2000	+11.6	CAMP factor

The table presents fold change in S phase in comparison with ML phase, classification into functional categories, and putative function.

**Table 2:** Putative CcpA binding sites in promoter regions of genes encoded by *S. agalactiae *NEM316 genome.

Match	Start (nt)	End (nt)	Homology with consensus(%)	ORF number	Name	Putative function
TGACAACGGTAAAA	16111	16124	92	gbsr001		16S ribosomal RNA

TGAAAACGCTTTAA	48894	48907	92	gbs0032		N-acetylmannosamine-6-phosphate 2-epimerase

TGACAAGGATGTCA	65156	65169	92	gbs0049	ruvB	Holliday junction DNA helicase ruvB

TTAAAGCGCTTTCA	69320	69333	92	gbs0053	adh2	Alcohol dehydrogenase

TGTAAACGATTACA	72238	72251	100	gbs0054	adhA	Alcohol dehydrogenase

TGGGAACGGTTTCA	130310	130323	92	gbs0119		ABC transporter permease protein

TGTAATCGCTTACT	130334	130347	92	gbs0119		ABC transporter permease protein

TATTAACGTTAACA	142634	142647	92	gbs0130		Membrane protease protein family

TGTCAACTATATCA	176297	176310	92	gbs0155		Multimodular transpeptidase-transglycosylase PBP 1B

TGTAATCGTTTACA	209972	209985	100	gbs0189		PTS system, trehalose-specific IIBC component

TGTAAACGGTTACT	214120	214133	92	gbs0191		Transcription antiterminator, BglG family

TGAAAAAGGTAACA	243786	243799	92	gbs0227		pseudogene

TGTTACCGTTTTCA	284183	284196	100	gbs0266		NADH peroxidase

TGAAAGCGGTTATA	349577	349590	92	gbs0326		Ribosome-associated factor Y

AGAAAGCGTTAACA	349601	349614	92	gbs0326		Ribosome-associated factor Y

TTAAAACGTTTTCA	375767	375780	92	gbs0348	manL	PTS system, mannose-specific IIAB component

TGATACCGTTCACT	480733	480746	92	gbs0452		alpha-L-Rha alpha-1,3-L-rhamnosyltransferase

TAATAACGTTAACA	515726	515739	92	gbs0493		Hypothetical protein

TGAAAACATTTACA	540267	540280	92	gbs0520	typA	GTP-binding protein TypA BipA

TGACACCGTTTTCA	592276	592289	100	gbs0569		Acetoin dehydrogenase

AGATAGCGGTCACA	608177	608190	92	gbs0583		Adenosine deaminase

TGATATCGCTTTCA	638255	638268	100	gbs0615		Class B acid phosphatase

TGAAAGTGTTGACA	661185	661198	92	gbs0644	cylX	Hypothetical protein

TAAAAGCGTTTACA	684988	685001	92	gbs0669		SUGAR SODIUM SYMPORTER

AGATAACGGTTACA	690270	690283	92	gbs0673		4-Hydroxy-2-oxoglutarate aldolase

TAAAAACGCTAACA	837159	837172	92	gbs0813		Glycerate kinase

TTAGAGCGTTTTCA	870536	870549	92	gbs0844	udk	Uridine kinase

TGTAAGCCTTGTCA	879217	879230	92	gbs0852		Hypothetical protein

TGTAAACCATCTCA	903332	903345	92	gbs0875	atpE	ATP synthase C chain

TGAAAACGTAATCA	903356	903369	92	gbs0875	atpE	ATP synthase C chain

TGTTAACGCTATTA	913902	913915	92	gbs0887	pheT	Acetyltransferase, GNAT family

TGAAAACCGTTTCA	981187	981200	92	gbs0940		16S rRNA m(2)G 1207 methyltransferase

TGAAAGCGTTTATA	1145634	1145647	92	gbs1100	pgmA	Phosphoglucomutase

AGAAAACGGTATCA	1157589	1157602	92	gbs1112	apbE	Iron-sulfur cluster assembly repair protein ApbE

TAATACCGTTATCA	1200221	1200234	92	gbs1156		Na+ driven multidrug efflux pump

TGAAATCGATTACA	1235422	1235435	100	gbs1192	gabD	Succinate-semialdehyde dehydrogenase [NADP+]

TGTAAAGGTTTTCA	1237447	1237460	92	gbs1195	ska	streptokinase

TGTAAACGTTTTTA	1248933	1248946	92	gbs1200		Hydrolase (HAD superfamily)

TTTAAACGCTATCA	1314589	1314602	92	gbs1273	rmlA	Glucose-1-phosphate thymidylyltransferase

TGAAACCGGTTTGA	1337103	1337116	92	gbs1293		glycerophosphoryl diester phosphodiesterase

TGAAAGCTCTGACA	1489796	1489809	92	gbs1437		Transcriptional regulators, LysR family

TGACAGCGCAATCA	1492240	1492253	92	gbs1441	capA	Capsule biosynthesis protein capA

TGTAACCGTTTTTA	1518448	1518461	92	gbs1468	pflC	Pyruvate formate-lyase activating enzyme

TGTAACCGCTTTCT	1742894	1742907	92	gbs1684		Zn-dependent alcohol dehydrogenase

TGTACACGATATCA	1749143	1749156	92	gbs1689		ABC transporter substrate-binding protein

TGAAAACCCTAACA	1752507	1752520	92	gbs1694		Dihydroxyacetone kinase

TGACAACGTTAAAA	1824783	1824796	92	gbs1764	mutS2	DNA mismatch repair protein mutS

TGTAAGCGTTTTAA	1920050	1920063	92	gbs1856	ulaA	PTS system, 3-keto-L-gulonate specific IIC component

TGACACCGGTATAA	1925222	1925235	92	gbs1862		Amino acid ligase family protein

TTATACCGTTTTCA	1929838	1929851	92	gbs1865	hslO	33 kDa chaperonin

TGTAAACGTTTTTA	1939040	1939053	92	gbs1874	ahpC	Peroxiredoxin

TGTAATCTCTTACA	1946899	1946912	92	gbs1875	ahpF	Peroxiredoxin reductase (NAD(P)H)

TTATAGCGCTTTCA	1957716	1957729	92	gbs1879	pepO	Oligoendopeptidase O

TGATAACTATGTCA	1990172	1990185	92	gbs1918	lacA.1	Galactose-6-phosphate isomerase lacA subunit

TGAAAGCGGTTTAA	2014283	2014296	92	gbs1939		PTS system, mannose fructose family IIA component

TGTAAACGCTTTTA	2101628	2101641	92	gbs2026	udp	Uridine phosphorylase

TGATATCGTAATCA	2130043	2130056	92	gbs2055	argR2	Arginine repressor, argR

AGATATCGCTTTCA	2157836	2157849	92	gbs2085		Ornithine carbamoyltransferase

AGAAATCGCTTTCA	2195324	2195337	92	gbs2122	arcA	Arginine deiminase

Genome was searched using BLAST algorithm (CLC DNA Workbench 4) with *cre *consensus sequence: TGWNANCGNTNWCA (N any nucleotide, W indicates adenine or thymine) and accuracy 90%. Start/End coordinates according to genome sequence

### Log phase related genes

Almost 50% of all GBS transcripts that were represented on the chip had similar patterns of expression and were classified into clusters 5, 10, 11, 12, 13, and 14. Transcript levels peaked in ML phase and decreased gradually to their lowest levels in S phase. These six clusters differ in their basal level of expression in L phase. The genes assigned to cluster 5 were expressed at low levels in ML phase, whereas genes in cluster 14 had very high transcripts in ML phase. Cluster 5 contains genes involved in multiple cellular and metabolic processes, whereas cluster 14 genes are involved predominantly in synthesis of ribosomal proteins. Clusters 12–14 contain genes encoding RNA polymerase subunits (gbs0084, gbs0105, gbs0156/7, gbs0302) that are down regulated from -2.3 to -10 times, which likely indicates a slowing of gene transcription. *RpoD *(gbs1496, encoding the major σ70) is also down regulated (~-3×). The RpoE subunit (gbs0105) plays a role in the development of sepsis during GBS infection [22], and its down regulation during growth might have consequences for GBS virulence.

### S phase related genes

We identified a group of known stress response genes present in clusters 1–4 that were significantly up-regulated in S phase, including *hrcA, grpE*, *dnaK *(gbs0094–96), *clpE*, and *clpL *(gbs0535 and gbs1376). Transcription of genes putatively involved in the stress response such as Gls24 and universal stress response family proteins gbs1202/1204, gbs1721, and gbs1778 were also elevated in S phase compared to ML phase (Table 1).

Two apparent operons responsible for arginine/ornithine transport and metabolism were also among the group of highly transcribed S phase genes. One operon (gbs2083–2085) encodes an arginine/ornithine antiporter, carbamate kinase, and ornithine carbamoyltransferase, respectively, and is up-regulated 350 to >1,000 times. The second operon (gbs2122–2126) encodes arginine deiminase, a second ornithine carbamoyltransferase, a second arginine/ornithine antiporter, and another carbamate kinase and is up-regulated ~55 to 150 times. Enzymes encoded by genes in these apparent operons are involved in arginine fermentation via the arginine deiminase pathway. They allow GBS to use arginine as an energy source after simple carbohydrates are exhausted from the medium, as would occur during stationary phase. On the other hand, activation of arginine deiminase pathway might have protective function against acidic conditions in a way similar to oral Streptococci [23] as we observed decrease of pH from about 7.9 to 5.5 between ML and S growth phases.

Metabolic changes toward the utilization of increasingly complex nutrient and carbon sources (see below) can be reflected by changes in utilization of simple carbohydrates (drop in the glucose concentration in the medium from ~300 mg/ml in ML to non detectable level in S) and by changes in transcription of the *glpKDF *(gbs0263–5, +45 to +63 times), a putative operon responsible for glycerol uptake and utilization.

### Trends in expression of genes involved in nutrient utilization and energy metabolism

**Figure 3:**
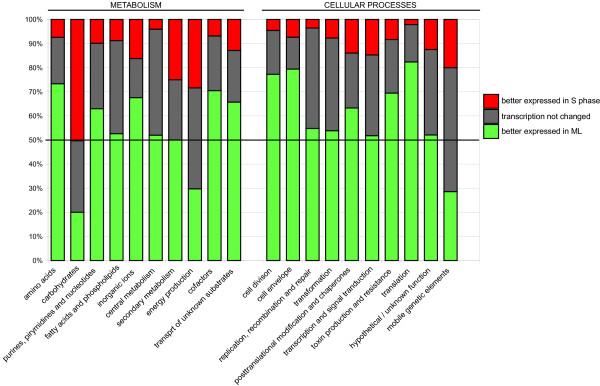
**Trends in transcript levels of genes involved in metabolism and cellular processes**. 1,994 of GBS transcripts represented on the chip were grouped into functional categories (see Table 1 and Additional file 2). The total number of genes in each category is shown as 100% and the number of transcripts more highly expressed in ML or S phase and transcripts with unchanged expression are presented as a fraction of the 100%.

In contrast to genes involved in other aspects of GBS metabolism and physiology, the only genes significantly up-regulated in S phase compared to ML were involved in carbohydrate metabolism (Figure 3). For example, we observed increased levels of certain glycolytic enzymes such as fructose-bisphosphate aldolase (gbs0125), glyceraldehyde 3P-dehydrogenase (gbs1811), phosphoglycerate kinase (gbs1809), enolase (gbs0608), pyruvate dehydrogenase (*acoAB*), and L-lactate dehydrogenase (gbs0947) (Table 1). This finding is similar to the results reported recently by Chaussee et al [19] showing that transcripts encoding proteins involved in carbohydrate utilization and transport were more abundant in S phase, presumably to maximize carbohydrate utilization. The authors suggested that increased transcription of genes involved in central metabolism and sequential utilization of more complex carbohydrates might be a particularly useful adaptation during infection of tissues where the concentration of carbohydrates is low [19]. In GAS, transcripts of genes involved in transport and metabolism of lactose, sucrose, mannose, and amylase were also more abundant during the stationary phase of growth [19], similar to our findings in GBS (Additional file 2). Similar to links between carbohydrate metabolism and virulence in GAS [21], also carbohydrate metabolism in GBS might be connected to strain invasiveness and strain tissue-disease specificity [24].

### Changes in expression of regulators and signal transduction systems

TCSs are especially important in the control of global gene expression, especially in the absence of alternative sigma factors. Of the multiple TCSs in GBS, only *covR/S *(gbs 1671/2) has been well characterized. CovR/S in GBS controls expression of multiple virulence factors, such as hemolysin, CAMP factor, and multiple adhesins [25]. The transcript levels of *covR/S *are down regulated in S phase, which may be responsible for the observed changes in transcription of virulence factors such as *cyl *genes encoding hemolysin. However, because the putative effect of CovRS on the *camp *and *cyl *genes seems to be opposite to those observed in *covRS *NEM316 mutant [26] it suggests that these genes are under influence of additional regulators.

Several other GBS genes encoding putative TCSs and regulators had significant changes in transcript levels during the growth phases studied. For example, transcript levels of gbs1908/9 increased 10/14 times between ML and S phases. The GAS homologs (M5005_Spy_0830/1 in strain MGAS5005 and Spy1106/7 in strain SF370) regulate an operon located downstream that encodes NAD-dependent malic enzyme and malate-sodium symport. In a ΔM5005_Spy_0830 deletion strain, the transcript of these downstream genes is decreased 23/40 times [12], indicating positive regulation. Organization of this chromosomal region in GBS is very similar to GAS, and gbs1906 and gbs1907 encode putative homologues to the GAS NAD-dependent malic enzyme and malate-sodium symport proteins, respectively. Genes gbs1906/7 are 63/81 times up-regulated in S phase; therefore this operon appears to be regulated in a similar manner in both GBS and GAS.

The transcript level of another GAS TCS homolog, gbs1934/5, is also elevated. Gbs1934/5 has close identity (~85%) with GAS M5005_Spy_0785/6 (Spy1061/2 in strain SF370), a TCS that has been implicated in the regulation of the mannose/fructose-specific phosphotransferase (PTS) system [12]. Interestingly, in GBS there is also a homolog of this PTS system located directly downstream of gbs1934/5 that is highly up-regulated (46.5 to 468 times) in S phase. Therefore, based on gene position, homology, and transcription regulation patterns, it is reasonable to speculate that these genes function similarly in GBS and GAS.

The possible functions of other TCSs can be inferred from their position. Two sets of TCSs are located directly upstream (gbs2081/2) and downstream (gbs2086/7) of an operon with arginine catabolism genes that are highly up regulated in S phase (see above). The transcript levels of both TCSs change dynamically during growth (Table 1 and Additional file 2). It is probable that genes encoding arginine catabolism proteins might be under tight control of both or either TCS. However, this needs to be confirmed experimentally. Thus, our transcript profiling results are consistent with the hypothesis that in the absence of global response gene regulation medicated by alternative sigma factors, GBS uses multiple TCSs as key mediators regulating the response to changes in the environment (Table 1).

Among putative regulators of unknown function, the highest changes were observed for gbs0191 encoding a transcriptional antiterminator of the BglG family (+50 times, putative CcpA binding site) and gbs0469 (-34 times). Surprisingly, we observed down regulation of expression of other global regulators that are associated with stress and the stringent response to starvation. These include the gene *relA *(gbs1928) that encodes a putative GTP pyrophosphokinase (-50), *codY *(gbs1719; -8), the cell density dependent regulator *luxS *(-3), and the putative *mecA *(gbs0135) homolog (-20). This result was unexpected given that *relA, codY*, and *luxS *are up-regulated in S phase GAS [19].

### Transcripts of proven or putative virulence genes

We observed changes in the transcript level of multiple genes encoding proteins with a carboxyterminus cell-wall anchoring motif. The putative location off the proteins on the cell surface suggests that they may play a role in GBS virulence or pathogen-host interaction. Four transcripts were significantly up-regulated in S phase gbs1420 (+6.3), encoding choline-binding protein, gbs1539 (+4.7) and gbs1929 (+5.5) encoding a putative nucleotidase, and gbs1143 (+2.6). We also observed down regulation in S phase of transcripts for several cell wall anchored proteins including a paralog of C5A peptidase precursor gbs0451 (-2), gbs1104 (-6.2), putative adhesin gbs1529 (-11) and fbp (gbs0850, -3), and putative laminin binding proteins (gbs1307, gbs1926; -3). Down regulation in S phase of proteins involved in bacterial attachment is consistent with results reported for GAS [14,15,19]. It is believed that several cell surface proteins are produced during the initial stages of infection to promote adhesion, and later are down-regulated to avoid immune detection.

Other known virulence factors of GBS that showed decreased transcription in S phase included an operon encoding hemolysin (gbs0644–0654), genes encoded on the putative pathogenicity island IX (gbs1061–1076), the putative group B antigen (gbs1478/9, gbs1481, gbs1484/5, gbs1492–1494), and genes involved in capsule synthesis (gbs1233–1247). The putative kinase *cpsX *(gbs1250) was upregulated 4.4 times (Table 1). Down regulation of capsule and putative and known surface antigens is known to occur in GAS [14,15,19]. For example, capsule, an antiphagocytic factor, is expressed during establishment of GAS infection and is later down-regulated once the infection is established [14,15]. Our results imply a similar scenario could be occurring in GBS. The only transcript encoding a proven virulence factor that was increased in S phase was CAMP factor (+11.6, *cfa*, gbs2000).

## Conclusion

Our results demonstrate that GBS gene transcript levels are highly dynamic throughout the growth cycle in vitro, likely reflecting exposure to an environment that is altering significantly during growth. The organism activates genes involved in metabolism of nutrients and carbon sources other than glucose such as complex carbohydrates and arginine and protect against changing pH. GBS slows down cell division and decreases transcription and translation. Production of virulence factors involved in establishment of the infection is reduced during growth. The global changes of transcript profiles we identified in GBS grown in rich medium are similar to patterns exhibited by GAS. Our results provide new information useful for the study of pathogen-host interactions and gene regulation in pathogenic bacteria.

## Authors' contributions

IS performed the research, IS and JMM analyzed the data and wrote the paper.

## Supplementary Material

Additional File 1 **Supplemental table 1- **Normalized hybridization values**.** File contains normalized hybridization values for each array used in the study. ML-mid logarithmic, LL-late logarithmic, ES-early stationary, S-stationary. P-"present" signal (detected in sample), M-"marginal" signal, A-"absent" signal (not detected). Click here for file

Additional File 2 **Supplemental table 2 **Changes in transcription of 1,994 transcripts present on array (S/ML ratio)**.** Green- genes down regulated in S phase, Red – genes up regulated in S phase, Gray – P values below 0.05. Click here for file
